# Essential evidence for guiding health system priorities and policies: anticipating epidemiological transition in Africa

**DOI:** 10.3402/gha.v7.23359

**Published:** 2014-05-15

**Authors:** Peter Byass, Don de Savigny, Alan D. Lopez

**Affiliations:** 1 Umeå Centre for Global Health Research, Department of Public Health and Clinical Medicine, Umeå University, Umeå, Sweden; 2 MRC/Wits Rural Public Health and Health Transitions Research Unit, School of Public Health, Faculty of Health Sciences, University of the Witwatersrand, Johannesburg, South Africa; 3 Department of Epidemiology and Public Health, Swiss Tropical and Public Health Institute, Basel, Switzerland; 4 University of Basel, Basel, Switzerland; 5 Melbourne School of Population and Global Health, University of Melbourne, Carlton, Australia

**Keywords:** health services, epidemiological transition, health information, sub-Saharan Africa, health policy

## Abstract

**Background:**

Despite indications that infection-related mortality in sub-Saharan Africa may be decreasing and the burden of non-communicable diseases increasing, the overwhelming reality is that health information systems across most of sub-Saharan Africa remain too weak to track epidemiological transition in a meaningful and effective way.

**Proposals:**

We propose a minimum dataset as the basis of a functional health information system in countries where health information is lacking. This would involve continuous monitoring of cause-specific mortality through routine civil registration, regular documentation of exposure to leading risk factors, and monitoring effective coverage of key preventive and curative interventions in the health sector. Consideration must be given as to how these minimum data requirements can be effectively integrated within national health information systems, what methods and tools are needed, and ensuring that ethical and political issues are addressed. A more strategic approach to health information systems in sub-Saharan African countries, along these lines, is essential if epidemiological changes are to be tracked effectively for the benefit of local health planners and policy makers.

**Conclusion:**

African countries have a unique opportunity to capitalize on modern information and communications technology in order to achieve this. Methodological standards need to be established and political momentum fostered so that the African continent's health status can be reliably tracked. This will greatly strengthen the evidence base for health policies and facilitate the effective delivery of services.

There is increasing evidence that, in many sub-Saharan African populations, death rates from major communicable diseases are declining, especially in childhood. As a result, countries are likely to advance through a process of epidemiological transition toward a greater burden of non-communicable diseases (NCDs), while still bearing a heavy communicable disease burden (1–5). It is much less evident that sub-Saharan African health systems or their health information sub-systems have adequate processes in place to adapt their systems and policies accordingly (6). Epidemiological transitions involve changes in patterns of births and deaths, and particularly in causes of death, and are inevitably accompanied by health transitions, in terms of the risks and diseases experienced, and changing patterns of health care needs. Measured trends in all-cause mortality suggest that patterns of morbidity and mortality are shifting, both in terms of cause and age distribution, with ensuing changes in therapeutic needs and demands (the most obvious example in sub-Saharan Africa [SSA] being anti-retroviral therapy against HIV/AIDS). Changing disease patterns are tending to increase prevalence compared with incidence for some key diseases (7, 8). Changing patterns of risk factors – whether in terms of vector exposure risks for infectious diseases or factors such as tobacco and alcohol consumption for NCDs – constitute a further critical component (9, 10). Long neglected health issues such as mental health are now increasingly seen as needing a health systems response (11). In low-income countries under-five mortality is decreasing at an impressive rate, albeit slower in SSA (12). These changes are occurring against a background of extremely scanty and often dubious data about what is actually happening (13). There is now, more than ever, a need to proactively update strategies for essential health data in SSA in order to increase the visibility of the continent's current and future population health trends and priorities.

It is unrealistic to expect that all SSA countries will achieve adequately high performance of national health information systems to global standards of timeliness, completeness, quality, and data use over the next 10–20 years. Thus, a transitional prioritised approach to improving the availability of critical health information in the short-term needs to be considered that is relevant to the essential policy actions that SSA countries must take now as the epidemiological transition unfolds. Currently, estimates of Africa's population health parameters tend to be made at the global level (14, 15), using such data as may be available as inputs to increasingly sophisticated models. The consequence of this is that national estimates for SSA countries tend to be a by-product of global estimates rather than contributing to them. This is completely counter to the principle of helping countries to strengthen their health systems through better health intelligence resulting from better information systems. Ideally, we need to move toward a model where a within-country data cycle becomes the normative source of information used locally and then fed into global estimates (Fig. 1), rather than *vice versa*. A typology of within-country data sources that might contribute to this is shown in Table 1.

**Fig. 1:**
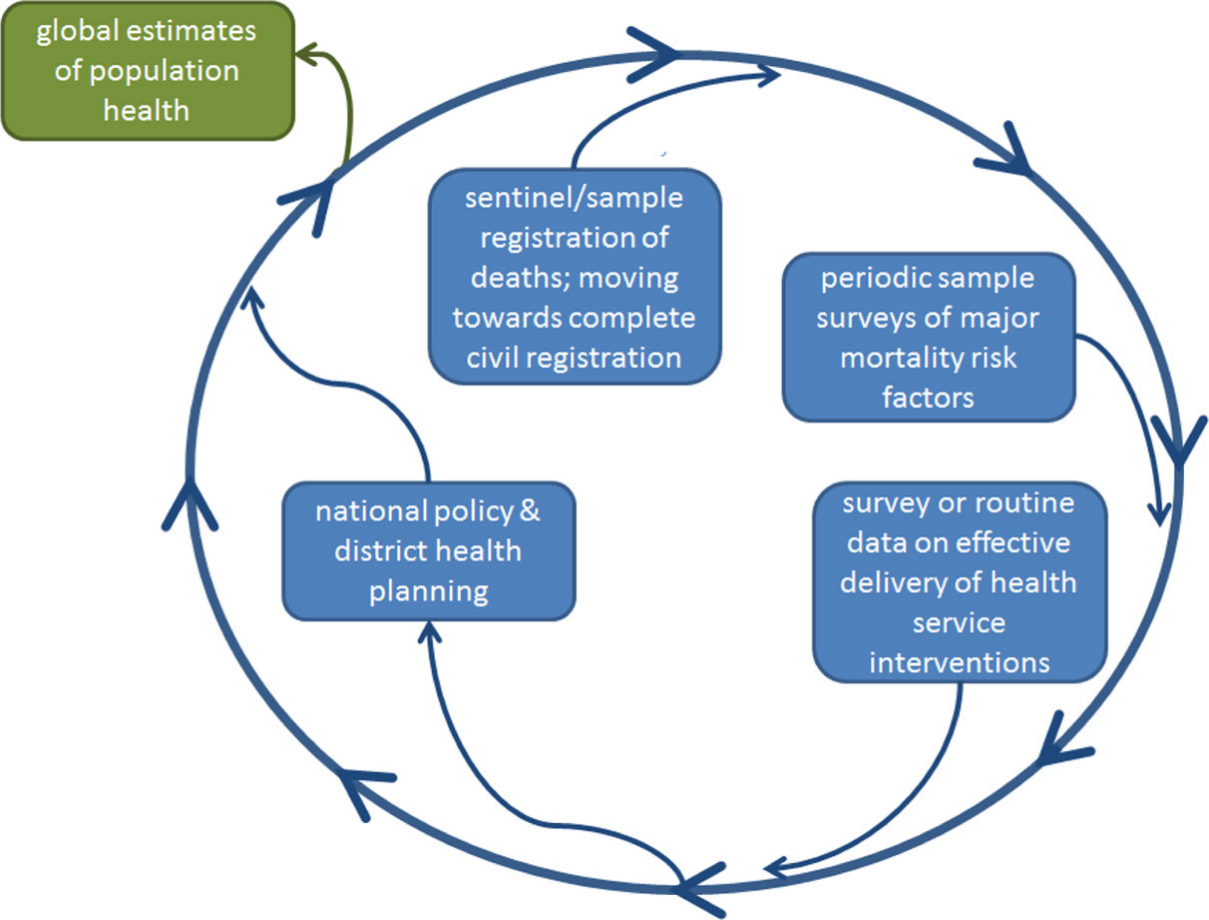
The concept of an in-country data cycle, also able to feed into global data.

The concept of health transition necessarily implies that not only health status but also rates of change need to be monitored and hence within-country data cycles need to be established on an on-going basis. This is a pre-requisite for ensuring a continuous supply of timely health information, rather than relying on a series of random, cross-sectional snapshots.

In this paper, we set out the principles of a minimum essential dataset that we believe all African countries should and could establish in order to guide health systems and policies through this epidemiological and health transition that is looming, and for which most sub-Saharan African countries are wholly unprepared. This paper is not intended to be an implementation guide but could inform the design of in-country data systems.

We propose that the essential components of such a minimum dataset are as follows:

1. continuous, reliable, unbiased *documentation of age- and sex-specific mortality, including the major causes of deaths* in the population (through routine civil registration with vital statistics, supplemented with sentinel or sample mortality surveillance systems with verbal autopsy [VA], where necessary);
2. biennial *documentation of exposure to the top 10 major risk factors for the leading causes of mortality* by age and sex (through population-based national household surveys); and
3. annual *documentation of the district-level coverage of key preventive and curative health interventions* for these major causes and risk factors for district planning purposes supplemented through a mix of periodic health facility assessments and surveys to determine full effective coverage of interventions for national policy purposes.

Some critically important strategic questions which need to be addressed in this context are:

1. *how* can these data sources be cost-effectively integrated within national health information systems to reliably describe the epidemiological transition dynamics for national populations?
2. *what* are the methodological implications for upgrading national health information systems to reliably measure epidemiological transition?
3. *which* ethical and political issues might drive long-term improvements in national health information in this direction?

We discuss each of these issues below.

1. **Documentation of age- and sex-specific mortality, including the major causes of deaths**.
  Complete and timely registration of all births and deaths at national level, including medical certification of cause of death, and collection of other key information about each birth and death, such as age of mother or age at death - for an entire population - is the optimal solution for monitoring epidemiological transition, as pioneered in Scandinavian countries and progressively implemented in other nations along with socioeconomic development (16). A circular issue arises, however, in that epidemiological transition normally accompanies other processes of population development, including the improvement of health information systems, and it is important not to misinterpret apparent longitudinal changes in population health that may actually reflect developments in its monitoring. It is equally clear that near-complete individual registration of births and deaths is not going to be widely implemented in SSA countries in the near future, for logistic and economic reasons, although this must be the primary goal, as elsewhere, of national health information development strategies (17). Therefore, it is important to consider what the ‘best-buy’ strategies for health data in SSA might be for the immediate decades, particularly emphasizing the need to reliably measure changes in health patterns over time. Representativeness of sub-national data is a crucial but difficult concept in this context. For reasons that are not always obvious, the frequent default assumption is that sub-national data are unrepresentative. This partly arises because it is very hard to demonstrate that any restricted set of data accurately represents a wider but unknown context. However, empirical evidence suggests that data may often actually be more generalizable than is thought to be the case. For example, in 1925, when Sweden was in many ways similar to a modern-day low- or middle-income country (LMIC), around 80% of counties had health indices closely comparable to national levels, meaning that most single counties chosen at random could each have adequately represented this relatively small country (18). This is an important consideration in the medium-term, when universal registration is not likely to be implemented in SSA countries. National estimates of proportional mortality show major similarities among neighbouring countries. In Tanzania, district burden of disease data have been used to inform district health priorities and resource allocation for other districts in nearby administrative regions (19).
  The overall choice of data sources therefore needs to combine a variety of sources, each with different strengths, which are complementary, and also each differently viable in a particular national context, but particularly considering the need to capture change over time. Factors such as the size and diversity of a country, the nature and coverage of its health system, local costs of relevant items such as wages, travel, communications, etc., and local history of more and less successful data collection strategies will also be important (20).
  A relevant resource in terms of practical steps toward tracking epidemiological transition has been provided by AusAID's Health Information Systems Knowledge Hub (http://www.uq.edu.au/hishub/) at the University of Queensland (17). Although this was designed primarily for Asian and Pacific countries, the principles translate well to SSA.
  Data sources for the first dataset on causes of death are several. Ideally, physician-certified causes of death incorporated into a civil registration system is the standard source of vital statistics to which all countries should aspire to develop and maintain. However, attaining adequate coverage of all deaths at national level (at least 90%) with sufficient quality of cause of death coding has proved elusive for low- and many middle-income countries and could potentially require decades to achieve, without concerted effort and resources. In the meantime, as civil registration and vital statistics (CRVS) systems slowly develop, the WHO and the former Health Metrics Network have recommended interim data sources (21). These are sentinel (urban & rural) demographic surveillance sites as a minimum, or where possible, more statistically representative sample registration sites, both with VA on all deaths in the sentinel or sample populations (17). These can be designed, funded, and implemented within 1–2 years and will produce useful longitudinal data thereafter on trends and dynamics in mortality by cause. Moreover, they will strengthen capacity both to produce and use cause of death data at country and sub-national levels. There is now extensive experience in implementing such sentinel mortality surveillance systems - Health and Demographic Surveillance Sites (HDSS) with VA - and growing experience in implementing sample registration with VA (SAVVY) (17, 22). VA methods for low- and middle-income countries are becoming increasingly standardized, adapted and simplified through machine coding of causes of death (23, 24).
2. **Documentation of exposure to the top 10 major risk factors of mortality**.
  Data sources for the second essential dataset on risk factor exposure can use standard adapted survey instruments for each risk factor (smoking, nutrition, high blood pressure, obesity, HIV sero-status, solid fuel smoke exposure, etc.) (25). However, these are rarely assembled into an omnibus national sample survey along the lines of the WHO STEPS (26). The Health Metrics Network and the Household Survey Network have been promoting greater integration and more strategic scheduling of national household surveys. Where this is done, the strategies should be reviewed to ensure that minimum indicators to inform epidemiological and health transition are included.
3. **Documentation of the effective coverage of key preventive and curative health interventions for these causes and risk factors**.
  Data sources for the third dataset on effective coverage of key health interventions targeting the top causes of death and risk factors are the least developed. Indicators of health service and intervention coverage and quality have the most sub-national (District) heterogeneity and can differ widely between neighbouring districts (27–29). District Health Information Systems have a long tradition of counting cases served (numerators). But there is a dearth of simple methodology that District Health Managers can apply to understand the actual annual reach and coverage of their own services, mainly due to missing denominators for many conditions. There is much room and need to innovate here by combining epidemiological and demographic information to provide districts with annual estimates of denominators (that is, likely cases of a particular disease to treat, size of target population for preventive services such as immunisation, etc.) to allow them to report and manage local coverage. In the meantime, coverage is usually only available from periodic cross-sectional national household or health facility surveys. This is one of the largest health information system weaknesses today (30). It is impossible strategically to manage district health systems without knowing effective coverage of key services (31). The key importance of this data source is it allows national health policy stakeholders to determine the alignment of their services and policies with population health needs.

## How can different sources of data be effectively integrated to yield a national picture of health transition?

Making connections between different data sources is always a major problem, particularly in countries where there is no universal unique personal identifier system. In countries with unique personal identification, it is possible in principle (subject to suitable ethical and confidentiality safeguards) to cross-link data of different types that relate to the same individuals (e.g. population-based data and health facility data) (32). However, that is not normally the case for SSA populations, and consequently there are very real difficulties in relating different data sources. SSA health facilities also tend to operate in very flexible catchment areas, with people often opting to consult out-of-area facilities because of perceived differences in issues such as quality of care and stigma, making links between population and facility data difficult. Consequently, connections between different data sources are very unlikely to be possible at the individual level, and even on a geographical basis (e.g. within districts) may be problematic.

**Table 1:** Typology of selected population and health facility level data sources potentially contributing to the specific national health information needs as described in this paper

Level	Model	Sample	Approach	Examples
National	National census	All	Complete cross-section	Most countries
	Civil registration with vital statistics	All	Complete longitudinal	Industrialized countries
	Sample registration or sentinel districts	1–2% of population	Longitudinal sample	China, India, Tanzania
	Cluster surveys	Cluster sample size	Repeatable cross-section	DHS surveys, WHO-SAGE
	Fixed panel surveys	Cohort sample size	Longitudinal cohort	Millennium Cohort Study
	Health facility surveys	All or sample of facilities	Self-selected group	Service availability and quality
Regional/Provincial	Complete population	All	Complete longitudinal	Universal registration
	Cluster surveys	Cluster sample size	Cross-section	Intervention coverage
	Individual surveillance	Defined area population	Complete in defined area	INDEPTH centres
District/local area	One-off or annual surveys	Survey sample size	Cross-sectional	Ad-hoc enquiries and district situation analyses
	Health facility surveys	All or sample of facilities	Self-selected group	Service availability, quality and use (for coverage numerators)
	Specific research	Context dependent	Specific issues of interest	Academic studies

**Fig. 2:**
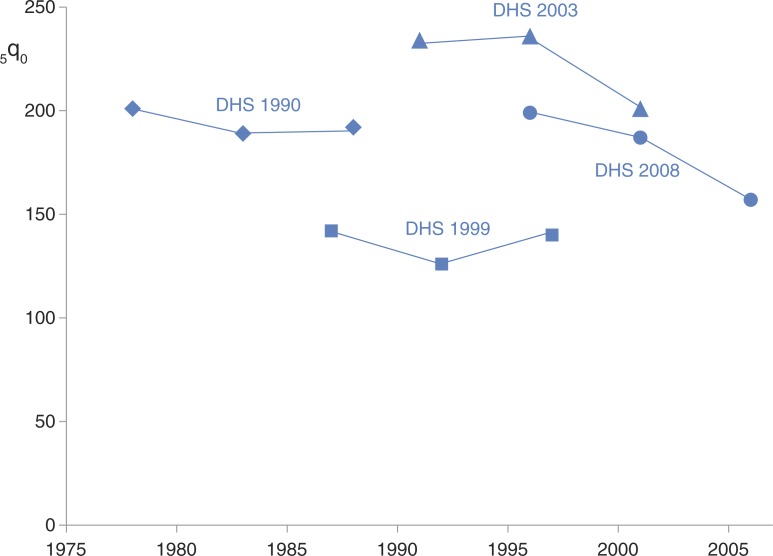
Under-5 child mortality (_5_q_0_) for Nigeria over three decades, as measured in four Demographic and Health Surveys (DHS).

The temporal component of any data intended to be used for understanding transition further complicates the issue. The example of the widely implemented Demographic and Health Survey (DHS) (33) and other national cluster sample surveys such as MICS (34), which typically draw a fresh cluster sample (up to around 10,000 households) at each 5-year interval, is important here. Although these surveys are designed to yield both time-of-survey cross-sectional data and retrospective data on factors such as mortality, the interpretation in terms of transition is complex because of the 5-year survey intervals. Repeated DHS surveys in a particular country permit pseudo-longitudinal approaches to analysis, but it has to be remembered that there is no intention in the DHS design to re-interview any individual or family longitudinally, meaning that issues of recall bias and inter-sample variation in these repeated cross-sections have to be considered carefully. Figure 2 shows under-five cumulative mortality for Nigeria, as estimated by four separate DHS survey rounds (35–38), which together make the interpretation of changes in this important index of epidemiological transition very difficult. Within the results from each survey, one might expect that increasing recall bias associated with deaths at longer periods before interviews (increasing recall effects at the left end of each survey line) could compete with temporal trends in decreasing mortality; from these four surveys it is difficult to interpret inter- or intra-survey trends over the 30-year period covered, even given the carefully controlled DHS methodologies used.

HDSS data from the INDEPTH Network (39) are an important source of detailed, longitudinal population data that are otherwise largely unavailable in SSA countries. INDEPTH centres typically cover geographically circumscribed populations (commonly between 50,000 and 150,000 people) longitudinally with regular household visits to record vital events and other data. It has been argued that these local, detailed data may constitute a more appropriate way of monitoring changes over time, such as is needed to monitor progress toward MDGs (40), although counter-arguments that such data may be unrepresentative are frequently made (4).

One possible way to use different sources of country data is to attempt cross-triangulations that compare outcomes across sources, although this can be difficult to accomplish in practice if comparable indices are not covered in different sources. One study from Mozambique compared census, HDSS and DHS data (41), suggesting that it should be possible to use all three sources synergistically in a national system. Several studies in SSA countries have made direct comparisons between national DHS surveys and HDSS data from specific areas within countries, though within-district numbers in DHS data are insufficient for local comparisons (42–44).

Household health survey programmes such as the DHS can add considerable value to national health information systems by providing timely and disaggregated data on mortality levels and trends. Recent advances in methodology to analyse survey responses to measure mortality levels and trends (45, 46) have greatly increased the utility of these methods by controlling for biases, particularly recall error and timeliness of measurements close to the date of the survey. Previously, the most recent estimates could only be made at least 2–3 years preceding the survey. Similarly, improvements to methods for measuring sibling survival have greatly increased the utility of survey-based responses for measuring levels and trends in adult survival.

## What would be the methodological implications for measuring epidemiological and health transitions in Africa in support of policy direction?

As health transitions eventually progress and possibly accelerate in SSA countries, it will become increasingly urgent to resolve the major challenges facing a reorientation of African health policies and systems from acute curative services to long-term chronic care. The fragile health systems in SSA, in many instances, have weak dynamic efficiency in terms of being able to respond to new developments (47). These challenges are mirrored for health information systems, which need to move beyond mainly documenting (in practice counting) acute episodes of illness, expanding in scope to include long-term chronic illnesses and repeated patient encounters and continuity of care. Clearly this is a more fundamental challenge than simply considering different diseases, since it implies a greatly increased need for reliable longitudinal follow-up, and in turn this indicates a more important role for longitudinal rather than cross-sectional data.

Accepting the premise that longitudinal data is an important component of information systems capable of tracking epidemiological transition, the additional resources required to implement such systems in most SSA countries would be considerable. Age and cause-specific death registration, at least within some defined population samples, is a pre-requisite for assessing epidemiological transition, and, in the absence of routine medically certified cause-specific death registration, this implies that introducing VA methods in a systematic manner is essential (24). Sampling considerations for population cause-of-death data are not trivial. As well as the conventional considerations of covering age and sex groups, assumptions need to be made about the smallest detectable cause-specific mortality fraction (CSMF). Since the size of ranked CSMFs in most populations follows an approximately exponential distribution, in effect this means deciding how far down the ranking it is worthwhile to go, and then sampling to make that *n*th rank measurable (48). Proof-of-principle for implementing VA methods, including probabilistic modeling of cause of death, with mobile technology (49) indicates that this is an approach ready to move out of research contexts and into routine usage for monitoring cause-specific mortality. Automated VA methods not involving physicians should be actively promoted as a major strategic component of any health information system for routinely tracking epidemiological transition in SSA since the performance of these methods has been found to be generally superior to physician interpretation of VA data, and are rapid, cheap, and consistent over time and place (50–52). One of the principal limitations to the widespread use of VA for measuring population cause of death patterns has been the non-standardized manner in which physicians interpret VA data, and their tardiness in doing so (53).

Less challenging are the national risk factor surveys from national household surveys. SSA countries routinely host repeated and uncoordinated, internationally funded, national household sample surveys such as the living standards surveys, household budget surveys, DHS and MICS surveys, malaria and HIV indicator surveys, and so on. The Health Metrics Network and the International Household Survey Network have encouraged greater integration, harmonization and synergistic scheduling of these surveys. Still there needs to be consensus methodology on the risk factor indicators and their methods of measurement to take advantage of these opportunities.

Where more innovation is needed is measuring both coverage (access) and full effective coverage (outcome) of essential health interventions aimed at the key causes of disease and injury burdens, including risk factors. There is a lack of practical methodology to do so, with the exception of a few interventions such as immunisation. For routine coverage estimates for district level planning, current Health Management Information Systems at local level do a good job of assessing numerators of the demand, but do not know the respective denominators for each service in the population at risk and in need of that service. Without this critical information, districts cannot estimate their local coverage, and therefore cannot plan and allocate local resources rationally. However without much effort, national programmes could provide expected prevalence or incidence figures from epidemiological surveys and other sources that could be coupled with district demographic data extrapolated from census data to provide the annual service related denominators. This type of epidemiological data sharing between national programmes and district implementers is feasible but practically never done. This is an easy step to determine a crude measure of service coverage (essentially access of those in need), which will already be a useful advance for district planning. Even this crude coverage can be useful for district planning because the rates monitored will often be well below what is expected. But this still stops short of determining effective coverage, which will be even lower than crude coverage. Innovation is needed for methodologies to determine the actual effective coverage of these essential interventions, which goes far beyond just access, to determining the quality and actual health outcomes of the interventions. Effective coverage is a measure of systems effectiveness and needs to be known periodically for policy purposes at national level across a sample of the health system. This will provide a barometer of the performance of the intervention in the real world health system concerned. Determining effective coverage requires a combination of survey and research effort across many disciplines in implementation science. Research and development is needed on these approaches and is already underway (54–56).

## Are there particular ethical and political considerations for measuring epidemiological and health transitions in Africa?

In countries with highly functional individual registration as the basis for all national information, including health, there generally exists a widespread (though perhaps not explicit) public confidence that the data system will operate in a confidential and ethical manner, and be under effective governmental regulation. However, it cannot be assumed that this is a readily transferable concept to other settings such as SSA countries. This may be a major barrier to expanding health information systems in SSA in acceptable ways.

While all individual health data should be handled within a robust framework of confidentiality, some data are understandably regarded as more sensitive than others. In SSA, the needs which have emerged for handling large numbers of HIV-related personal records and test results have to some extent brought requirements for patient confidentiality into focus, in contrast to the past when local health centres tended to be very casual about such matters. This demonstrates that paradigm shifts in practice are possible.

There are also important ethical and political considerations surrounding the use of outputs from an effective health information system. In one extreme example, a maternity facility in Burkina Faso was burnt down by protesters angry about the alleged neglect of a woman who died in labour, illustrating that information about health outcomes is by no means a neutral commodity (57), and carries implications for accountability.

National governance issues also impinge on the organization of health services and hence health information systems. In larger countries, operating on a federal or quasi-federal basis with considerable regional autonomy, it may be more appropriate to focus efforts on running good information systems at the regional level. Individual regions in larger countries may be larger than small countries. If health information systems operate well at regional level, then aggregating to national level should be simple. Nigeria and Ethiopia, the two most populous countries in SSA, would be key examples of countries where regional approaches would be valuable.

## Moving forward to improve understanding of epidemiological transition in SSA

The current scenario where the health information map of Africa is largely characterized by empty spaces is manifestly unacceptable in today's information age and given the critical need for essential information support to health policy development in Africa. This deplorable situation requires urgent action. Every SSA country needs encouragement and technical resources to improve health information systems to the point where at least reliable in-country national estimates of key parameters become available. Because health in SSA countries is increasingly dominated by longer-term diseases and conditions, good national estimates must have a longitudinal basis and thus be able to reflect change reliably. Sub-national data are also critically important for proper national planning, especially in larger countries, wherein wider variations in health and population exposure to risk factors might be expected. Timeliness of data, particularly on leading causes of death and how they are changing is also critically important if the data are to be useful to inform policy.

For locations where, in the medium-term, universal individual registration of births and deaths and medical certification of cause of death is not feasible, it is strategically important to integrate established population surveillance sites into national data systems, replicating such sites where appropriate. This implies moving from an isolated field site model of surveillance toward sentinel site networks, such as have been implemented successfully in India and China (58, 59). A balance needs to be struck among emphases on measuring mortality, morbidity and risk-factor outcomes.

A strategic focus is needed such that resources and effort are not dispersed or duplicated over all elements of a health information system, but targeted on the critically important elements of health system management. As argued in this paper, these are: 1) mortality (by age, sex and cause), but not morbidity; 2) periodic data on population-level exposures to major (selected) risk factors, particularly those important for NCDs and injuries; and 3) some measure of the health system response to these health threats, especially the effective coverage (as opposed to measured or estimated coverage) of essential health interventions against the major causes of disease burden.

Clearly these are not strategies that can be implemented without effort or cost. Human resources, particularly in relation to some of the technical issues of data management and quality control, are very scarce in some SSA countries, especially in the government sector. Therefore, careful national implementation plans need to be worked out that are contextually appropriate. There will undoubtedly be implementational challenges; however, the question must be asked whether SSA, and indeed the world, can continue to drift without effective and actionable information on the health of 856 million of the 6,895 million (12%) of the world's population (60). Since this proportion is projected to rise to 1,960 of 9,306 million (21%) by 2050, any delay in tackling the problem will only increase the magnitude of the difficulties.

## Conclusions

A more pro-active and strategic approach to health information development in SSA is urgently required by the international community. Local policy is likely to be more effective when based on local evidence. This implies a shift away from relying on global comparative exercises that generate sets of national estimates toward facilitating the development of disaggregated within-country health information systems that are targeted toward monitoring both the leading causes of ill-health in populations, and the response of the health system to controlling them. Lessons must be learnt from effective systems that have been implemented in other regions, but adapted to the African context so that effective data linkages can be made across various levels of health systems and corresponding populations. SSA countries have a chance to take full advantage of the ICT revolution and leap ahead in the design of efficient and interoperable data collection, integration and dissemination technologies in e-Health and m-Health (61). A culture of using health information critically and openly for steering new health policies and system strengthening needs to be encouraged and fostered, and this demands the availability of timely and reliable health statistics.

Health status in some low-income SSA countries is changing more rapidly now than at any prior time in history. To track and steer these population health dynamics and understand what such transitions mean for health systems and policies requires radical change and strengthening of national health information systems to provide essential information. What we propose in this paper is concerted investment on three fronts:

1. interim, strategic investments in sentinel or sample registration systems that provide timely, quality, longitudinal data on deaths and causes of deaths while developing effective civil registration systems for vital statistics;
2. periodic national cross-sectional omnibus sample surveys of the top 10 major risk factors for the leading causes of death; and
3. development of new approaches to estimating district level intervention coverage (access) for annual district planning purposes, and for periodically estimating full effective coverage for national policy purposes through better combinations of routine health service statistics, demographic and epidemiological data for the essential health interventions relevant to these causes.

Countries in SSA and their international funding partners and the global health research community need to prioritize these dimensions in their approach to national strategies for health information systems strengthening. However, information without an appropriate health policy and health system response is not enough. The strengthening of the health information system in this direction needs to be coupled with health system strengthening in order to respond effectively to the dynamics imposed by the health transition (6, 62–65).

The main impediment to implementing these three re-directions of the Health Information System is presently methodological. We need innovations in monitoring burden of cause-specific mortality; in monitoring its attendant risk factors; and in monitoring and verifying the actual coverage of required health system responses. These innovations should focus on improving: sentinel and full CRVS approaches; national household risk factor survey methodologies; and practical coverage assessment methods that can be implemented at, and by the district level.

There is growing momentum toward Universal Health Coverage in the Post-2015 Sustainable Development Goals. These goals will take greater cognizance of demographic change and the social determinants of health (66). The data collection platforms and priorities proposed in this paper will be essential for understanding universal coverage and how it can be attained. The time is right to consider a proactively deliberate updating of strategies for essential health data in SSA in order to more reliably understand the continent's current needs to improve population health and to better prepare for future trends.

**Main findings**

- Although epidemiological transition is generally considered to be underway in sub-Saharan Africa, health information systems in most places are insufficient for adequately tracking developments in population health, leading to major gaps in the knowledge needed to plan effective health services.
- Many countries in SSA get key national health indicators from global estimation processes, rather than gathering adequate national data to understand their own situation and feed into the construction of global estimates; this is insufficient for health planning.
- Very few sub-Saharan African countries currently have or are close to having effective systems for national universal civil registration and vital statistics (CRVS; registering all citizens, including births, deaths and cause of death, and using those data effectively in national statistics).

**Key messages for action**

- Most sub-Saharan countries are unlikely to implement complete death registration with physiciancertified cause any time soon. Although this should be the long-term aim, interim solutions must use standardised verbal autopsy procedures with automated cause of death assignment.
- A consensus is needed on a minimum essential dataset to underpin effective national health information systems in sub-Saharan Africa. Key components would include complete civil registration including verbal autopsy, populationbased documentation of major risk factors, and documentation of health service coverage for key preventive and curative measures.
- A key priority across sub-Saharan Africa is the investment in national-level systems that can gather and handle health information effectively, including procedures, equipment and human resources. Countries and their international partners need to understand health status on a population-wide basis.
